# Study design and baseline findings from the progression of ocular findings (PROOF) natural history study of dry eye

**DOI:** 10.1186/s12886-017-0646-5

**Published:** 2017-12-28

**Authors:** Peter J. McDonnell, Stephen C. Pflugfelder, Michael E. Stern, David R. Hardten, Taryn Conway, Linda Villanueva, David A. Hollander

**Affiliations:** 1 Wilmer Eye Institute, Johns Hopkins University School of Medicine, 600 N. Wolfe St., Maumenee 727, Baltimore, MD 21287-0005 USA; 2 Baylor Eye Physicians & Surgeons Houston, 6565 Fannin NC 205, Houston, TX 77030 USA; 3 Allergan plc, 2525 Dupont Drive, Irvine, CA 92612 USA; 4 Minnesota Eye Consultants, 710 E 24th St, Minneapolis, MN 55404-3840 USA; 5 Jules Stein Eye Institute, 100 Stein Plaza, University of California, Los Angeles, Los Angeles, CA 90095 USA; 6 Present address: 14 Altezza Drive, Mission Viejo, CA 92692 USA

**Keywords:** Dry eye disease, Natural history, Tear break-up time, Cytokines, Tear film biomarkers

## Abstract

**Background:**

The aim of this research is to initiate a 5-year natural history study of dry eye disease (DED) using objectively assessed and patient-reported outcomes, to explore the hypothesis that DED is a progressive condition that has substantive and measurable impacts not only on the ocular surface, but on quality of life and visual functioning. Our objective for this report is to examine the baseline data.

**Methods:**

A multicenter, prospective, controlled, observational study of Level 2 (mild-to-moderate) DED patients based on International Task Force Delphi Panel severity grading, and controls, documented baseline measures (including tear film biomarkers and quality of life). Tear cytokine concentrations were also measured in the tear film. Patients were using artificial tears as needed.

**Results:**

Two hundred seventeen DED patients and 67 gender- and age-matched controls were enrolled. A majority were females and Caucasian and groups did not differ significantly in terms of gender, race, or age. Differences between DED and matched controls, at baseline, included mean scores for Ocular Surface Disease Index (31.7 vs 4.1, *P* < 0.0001), Schirmer test (5.7 vs 15.3 mm, *P* < 0.0001), corneal staining (1.4 vs 0.2, *P* < 0.0001), conjunctival staining (1.4 vs 0.3, *P* < 0.0001), and tear break-up time (5.7 vs 8.5 s, *P* < 0.0001). Tear cytokines levels were determined and included interferon-γ, interleukin (IL)-1β, IL-2, IL-4, IL-6, IL-8, tumor necrosis factor-α, epidermal growth factor, IL-13, IL-17, IL-1α, and inducible protein-10. The mean levels of IL-8 and IL-6 were slightly higher in the DED group at baseline. Blurred vision was reported as moderate/severe/very severe at baseline in 57.6% of DED patients vs.10.5% of normal controls (P < 0.0001). DED patients reported greater reductions in work and non-work productivity, as well as greater need for visits to ophthalmologists during the prior year.

**Conclusions:**

In this report of the baseline findings of a 5-year natural history study of DED, a striking disease burden is observed with regard to blurred vision, productivity, and visits to eye care practitioners in mild to moderate DED patients compared to normal subjects of similar ages and genders.

**Trial registration:**

ClinicalTrials.gov NCT00833235 on January 30, 2009.

## Background

Dry eye disease (DED) ranks amongst the most common disorders impacting the adult population and is one of the leading reasons for patient visits to eye care specialists in the US [1]. Despite the prevalence of DED, our understanding of numbers affected and the natural history of the disease is severely limited. There has been one study that focused on patient self-reports of DED severity over time, but this study did not examine the impact of specific inflammatory cytokines identified in the tear film of patients on the progression of DED [2]. There are few data on DED due to lack of any single diagnostic to serve as the “gold standard” to reliably distinguish affected from normal and the common lack of correlation between objective signs and symptoms of the disease.

It is estimated that about 5 million Americans (3.2 million women, 1.7 million men) aged 50 years or older are afflicted with DED [3, 4]. Large epidemiological studies report a range from 5 to 35% [5, 6] in varying age groups. By comparison to other relatively common ophthalmic diseases, the attention paid in peer-reviewed ophthalmic literature to DED is low in proportion to the number of individuals affected. According to The Dry Eye Workshop, “tens of millions more [Americans] have less severe symptoms and probably a more episodic manifestation of the disease” [7]. Widely accepted amongst clinicians, this latter statement implies that DED is second only to refractive error as the major source of ocular morbidity in developed countries like the United States.

Once considered to represent an involutional component of the normal aging process, especially in women, DED is now thought to represent localized autoimmune disease originating from imbalances in protective immunoregulatory and proinflammatory pathways of the ocular surface [8]. While the exact etiology and pathogenesis of the condition remain unclear, a number of inflammatory mediators have been reported to be increased or decreased in DED patients compared to normals [9].

Currently, cyclosporine 0.05% ophthalmic emulsion (Restasis, Allergan plc, Dublin, Ireland) and lifitegrast 5% ophthalmic solution (Xiidra, Shire, Lexington, MA, USA), are the only Food and Drug Administration-approved prescription therapies for DED [10, 11]. Cyclosporine 0.05% ophthalmic emulsion is approved for the indication of improving tear production in patients with keratoconjunctivitis sicca with reduced tear production due to presumed inflammation. In a registry of patients with Sjogren’s syndrome, an autoimmune condition associated with elevated risk of DED advanced-stage development, a large proportion (85%) of patients had symptoms of DED, with 43% having those symptoms for longer than 5 years, yet only 8% were being treated with the approved cyclosporine ophthalmic emulsion [12]. Similarly, in a health claims database study of 576,000 patients, only 14% of DED patients filled at least one prescription for topical cyclosporine during a 2-year study period. When other prescription therapies were included in the analysis that are not specifically approved or indicated for DED, a total of 38.5% of patients filled a prescription for topical cyclosporine, a topical corticosteroid, or an oral tetracycline [13]. Thus, it appears that the vast majority of DED patients are not being treated with prescription medications for their condition, in particular medications targeting the underlying inflammatory etiology. These findings suggest that there may be limitations in the diagnosis and classification of dry eye severity, perceptions of limited efficacy, or an unfavorable/risk/benefit profile with anti-inflammatory pharmacotherapies in DED. Another possible explanation for these findings is the uncertainty about the natural history of DED and, by extension, the degree of improvement that is expected from prescription therapeutic interventions.

Lack of knowledge of natural history of DED makes appropriate counseling and management of patients problematic. In DED studies to date, the control group has been assigned to a special regimen that typically includes an unpreserved vehicle formulation that may include emulsions that are quite distinct from the preserved topical lubricating drops that DED patients typically purchase without a prescription and use in managing their conditions. In addition, compliance of subjects in clinical trials is known to vary markedly from that of patients outside of studies. Consequently, data generated from control arms of clinical trials may not necessarily represent the natural history of DED.

A major limitation in our understanding of DED relates to the impact of specific inflammatory cytokines identified in the tear film of patients on the progression of DED. The current study was designed to determine rate of progression of DED in a large natural history study and to determine the predictive value of baseline clinical measures, including biomarkers, in predicting disease behavior in individual patients over time. As part of this project, baseline measures of patients and controls (without disease) revealed important characteristics of the manifestations of DED in a large population. The study is ongoing and we now report the patient baseline data from the PROOF study.

## Methods

### Study design

**Fig. 1:**
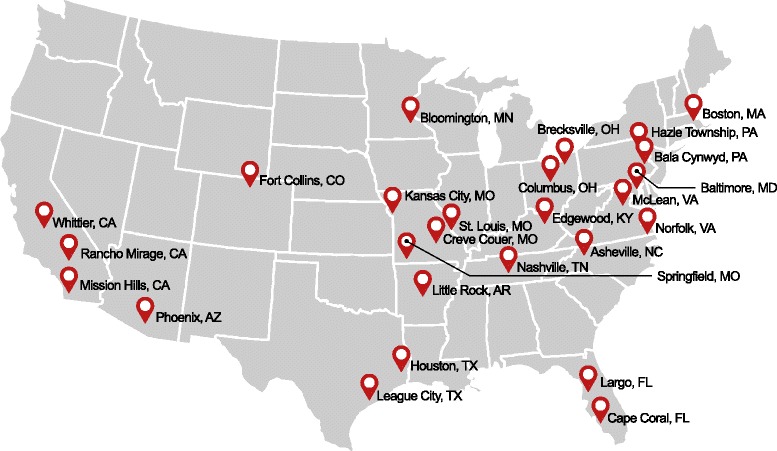
Geographic location of clinical investigators

This is a multicenter (15–20 geographically diverse sites in the United States; Figure 1), prospective, controlled, observational 5-year study of the natural history of patients with DED. This study was approved by the Western Institutional Review Board and institutional review boards at Mercy Health System Springfield, Johns Hopkins University, Vanderbilt University, University of Arkansas for Medical Sciences, and Baylor College of Medicine and Affiliated Hospitals. The study was discussed with the patient and a participating patient gave Authorization for Use and Release of Health and Research Study Information, and other written documentation in accordance with the relevant country and local privacy requirements. Each enrollee providing informed consent was assigned a unique four-digit identification number to be used on patient documentation throughout the study. The procedures used in this study adhered to the tenets of the Declaration of Helsinki.

Patients were not randomized, but approximately 200 patients with  DED and 50 gender- and age-matched controls (without evidence of chronic DED) were enrolled. The first and last patient recruitment dates were 10th September 2009 and 20th November 2013, respectively. As this is a natural history study, the expectation at enrollment was that patients would continue with any artificial tear therapy they had already elected to use to manage their symptoms. Therefore, no physician advice or recommendations to modify therapy were given to subjects, and patients continued with any artificial tear regimen they were using as needed. There were 12 scheduled visits during the study: Screening (Day −7), Baseline (Day 0), Month 6 (Day 182 +/−14), Month 12 (Day 365 +/− 14), Month 18 (Day 547 +/− 14), Month 24 (Day 730 +/− 14), Month 30 (Day 912 +/− 14), Month 36 (Day 1095 +/− 14), Month 42 (Day 1277 +/− 14), Month 48 (Day 1460 +/− 14), Month 54 (Day 1642 +/− 14), and Month 60/Exit (Day 1825 +/− 14). The baseline screening has been completed and forms the basis of this report.

### Inclusion criteria

Key inclusion criteria included males aged at least 55 years or females aged at least 40 years who are either postmenopausal or with perimenopausal symptoms of at least 6 months’ duration, best-corrected visual acuity (BCVA) in at least one eye of 20/80 or better, ability to provide written informed consent prior to commencing any study-related procedures, and willingness and ability to comply with the investigator and protocol instructions with a high likelihood of completing all required visits.

#### Additional inclusion criteria for DED patients

Additional inclusion criteria for DED patients included baseline Ocular Surface Disease Index (OSDI) score of >13 (based upon a 0–100 scale) and Level 2 International Task Force (ITF) Delphi panel score on ITF Delphi Panel [14] (moderate symptoms, presence of conjunctival staining and of corneal staining [grade > 1 on Oxford Scheme] without central staining), and Schirmer’s test with anesthesia <7 mm.

#### Additional inclusion criteria for control subjects

Additional inclusion criteria for control subjects included baseline OSDI score of <8 (based upon a 0–100 scale), Schirmer’s test (with anesthesia) >10 mm, and BCVA in at least one eye of 20/80 or better.

### Exclusion criteria

#### DED patients

Key exclusion criteria for DED patients included satisfying criteria for ITF categories 1, 3, or 4 (signs such as central corneal staining and filamentary keratitis), Sjogren’s syndrome, use of topical cyclosporine within 3 months of baseline, history of herpes keratitis or varicella zoster keratitis, any history of allergic conjunctivitis, temporary or permanent occlusion of the lacrimal puncta of either eye, use or planned use of topical glaucoma medications during the study, anterior segment surgery involving a limbal or corneal incision (e.g., cataract surgery), keratorefractive procedure (e.g., laser in situ keratomileusis, laser epithelial keratomileusis, photorefractive keratectomy) or trauma that could have an impact on corneal sensitivity within the last 12 months or expectation of such surgery during study period, history of penetrating keratoplasty, current use and/or past use within 2 weeks of baseline and/or anticipated use during the study period of any topical ophthalmic steroid or nonsteroidal anti-inflammatory agent, current use of and/or use within 2 weeks of baseline of any oral tetracycline or tetracycline-like product (e.g., doxycycline) or topical macrolides (azithromycin), current use within 2 weeks of baseline of any oral or topical nutritional supplements (e.g., flaxseed oil), presence of a condition that in the investigator’s opinion, may put the patient at significant risk or confound the study results, or may interfere significantly with the patient’s participation in the study, and current enrollment in any other clinical study or participation in such a study within 30 days of entry into this study.

#### Exclusion criteria for control subjects

Additional exclusion criteria for control subjects included regular symptoms of DED or the presence of corneal or conjunctival staining.

### Outcome measures

Measures of patient and control subject outcomes in this study are in Table 1. Baseline differences between patients and control subjects were assessed for corneal (with fluorescein) and conjunctival staining (with lissamine green) using the Oxford 0–5 scale at 1 unit increments, Schirmer’s test (with anesthesia), OSDI score (scale 0–5), tear break-up time (seconds), and reports of blurred vision (using a 5-point scale, 0–4. Goblet cell densities of the inferior bulbar and temporal bulbar regions areas of the conjunctiva were measured by impression cytology, and tear film break-up time and tear cytokine concentrations were recorded at baseline using techniques as previously published [15, 16]. Tear cytokines measured included interferon (IFN)-γ, interleukin (IL)-1β, IL-2, IL-4, IL-6, IL-8, tumor necrosis factor (TNF)-α, epidermal growth factor (EGF), IL-13, IL-17, IL-1α, and inducible protein (IP)-10. The visits and procedures to take place are summarized in Table 2. Impression cytology specimens were examined for adequacy. If no cells were present, the sample was interpreted as being of poor quality or that cells were not picked up, and that specimen was not included in the analysis.

**Table 1:** Outcome measures for dry eye disease (DED) patients and control subjects

Efficacy measures	
Primary:	The percentage of patients with signs of DED progression based on at least 1 level increase in severity level as measured by ITF guidelines. Evidence of disease progression by ITF guidelines will require at least one of the following: • 1-unit increase in total corneal staining • New development of central corneal staining (≥ grade level 1) • Increase in OSDI score by ≥6 points • Increase in blurred vision ≥1 grade (using a 5-point scale)
Secondary:	• % of patients who are discontinued from the study because they need a therapeutic intervention (i.e., the use of cyclosporine, punctal plugs, oral tetracyclines, topical steroids, topical antibiotic ointments, or oral nutritional supplements) due to dry eye disease progression • % of patients with a ≥ 6 point OSDI increase • % of patients with a 1 unit increase in blurred vision • % of patients with a 2 mm decrease in Schirmer’s test over 5 min • % of patients with at least a 1 grade increase in central or total corneal staining • Reduction in reading speed
Other:	• Change from baseline in goblet cell density (by impression cytology) • Tear Break-up Time • Tear Cytokines • Dry Eye Patient Survey
Safety Measures	• Adverse Events, Biomicroscopy, BCVA

**Table 2:** Summary of baseline assessments and procedures in order of conduct

• Inclusion criteria and authorization	
• Medical and ophthalmic history	
• Concomitant medications review	
• Adverse event review	
• OSDI questionnaire	
• Dry Eye Patient Survey	
• Blurred/Fluctuating Vision Assessment	
• Work Productivity and Activity Impairment Questionnaire	
• Best-corrected visual acuity	
• Reading Speed Assessment	
• Tear collection	
• Biomicroscopy	
• Tear break-up time	
• Corneal staining (with fluorescein)	
• Conjunctival staining (with lissamine green)	
• Schirmer’s test (with anesthesia)	
• Goblet cell density (by impression cytology)	
• Dispense patient diary	

The clinical sites received a procedure manual and accompanying video detailing proper technique for tear collection. In brief, as we previously described [16], the clinical sites were instructed to collect 1 μL of tears by placing a graduated 1 μL capillary tube in the inferior temporal cul-de-sac, taking care to avoid contact with the epithelium and reflex tearing. Following collection, 1 μL of tears was eluted into the bottom of the Sarstedt tube containing 9 μL of cytokine assay buffer, yielding a final volume of 10 μL; select cytokines were measured using multiplex bead analysis and total concentration extrapolated using a dilution factor of 1:10 (1 μL tears +9 μL buffer). Only samples that adhered to the standard procedures detailed in the procedure manual, assessed by a final sample volume of 10 μL, with a range of ±1 μL to allow for a small degree of pipetting error were accepted for analysis. Following each run, clinical sites received feedback on the accuracy of the sample volumes so that they could take action, if appropriate, to more accurately achieve the target volume of 10 μL.

### Statistical methods

Description of baseline characteristics included summary statistics for continuous and categorical variables. For continuous variables the sample size (N), mean, standard deviation (SD), median, minimum (min), and maximum (max) were presented. For categorical variables the number and percentage of subjects were presented. Statistical tests were used to compare baseline findings between the control and all-cases group. All *P*-values are 2-sided and considered to be statistically significant at the α ≤ 0.05 level.

The proportion of control patients showing disease progression was estimated to be so small that it was considered impractical to include an adequate number of patients as a control arm. The primary analysis was based upon different hypothetical rates in a control population. For example, a one group chi-square test with a 0.050 two-sided significance level would have 85% power to detect the difference between the null hypothesis proportion of 0.020 and the alternative proportion of 0.060 when the sample size is 170. Assuming a 15% dropout rate, approximately 200 cases were required to be enrolled into this study to provide 170 cases with data at the end of 60 months. No adjustments for multiplicity were planned.

## Results

### Demographics of enrolled patients and control subjects

Patients and control subjects were enrolled into the study by a group of geographically diverse study investigators practicing in academic as well as private practice settings (Fig. 1). A total of 217 patients with mild-to-moderate DED and meeting the inclusion and exclusion criteria were enrolled, as were 67 normal controls. In all, 2–30 patients were enrolled per site in the Eastern states, 1–34 were enrolled per site in the Midwest, and 17–25 were enrolled per site in the West. This represented an equal distribution of subjects across climatically diverse regions. A majority of enrollees were females and Caucasian. The patient and normal subject populations did not differ significantly in terms of gender, race, or age (Table 3).

**Table 3:** Summary of patient demographics at baseline: dry eye disease versus control populations (all enrolled population)

	All cases (*n* = 217)	All controls *(n* = 67)	*P*-value
Gender			
Male	40 (18.4%)	16 (23.9%)	0.3797
Female	177 (81.6%)	51 (76.1%)	
Race			
Caucasian	186 (85.7%)	59 (88.1%)	0.4000
Black	12 (5.5%)	3 (4.5%)	
Asian	0 (0.0)	1 (1.5%)	
Hispanic	18 (8.3%)	4 (6.0%)	
Other	1 (0.5%)	0 (0.0%)	
Age (yrs)			
Mean (SD)	63.2 (10.5)	61.1 (11.2)	0.1607
Min–Max	37–89	41–88	

DED patients had significantly worse (*P* < 0.001) scores than normal controls in mean (SD) corneal staining score (1.4 [0.9] vs 0.2 [0.5]), conjunctival staining (1.4 [1.0] vs 0.3 [0.6]), Schirmer’s test score (5.7 [4.2] vs 15.3 [7.3] mm), OSDI (31.7 [19.6] vs 4.1 [7.4]), and tear break-up time (5.7 [4.0] vs 8.5 [5.0] s) (Table 4).

**Table 4:** Measures of signs and symptoms at baseline in dry eye disease and control populations (all enrolled population)

	All cases (*n* = 217)	All controls (*n* = 67)	*P*-value
OSDI Score			
Mean (SD)	31.7 (19.6)	4.1 (7.4)	<0.0001
Min-Max	0.0–91.7	0.0–39.6	
Schirmer’s score (mm)			
Mean (SD)	5.7 (4.2)	15.3 (7.7)	<0.0001
Min-Max	0.0–30.0	1.0–35.0	
Corneal staining score			
Mean (SD)	1.4 (0.9)	0.2 (0.5)	<0.0001
Min-Max	0.0–4.0	0.0–2.0	
Conjunctival staining score			
Mean (SD)	1.4 (1.0)	0.3 (0.6)	<0.0001
Min-Max	0.0–5.0	0.0–2.5	
Tear break-up time(s)			
Mean (SD)	5.7 (4.0)	8.5 (5.0)	<0.0001
Min-Max	1.0–28.3	1.3–25.0	

Although impression cytology in DED enrollees revealed a mean goblet cell density of the temporal bulbar conjunctiva about 19% less than controls, the difference was not statistically significant (Table 5). No differences in the inferior bulbar region were found between the patients and controls. There was a statistically significant lower goblet cell density in the temporal region relative to the inferior region in the DED patients. There was no difference between the temporal and inferior regions in goblet cell density in the control group. Comparison of DED enrollees and normal subjects according to the symptoms they experienced (Table 6) revealed that for all specific symptoms (stinging/burning, dryness, itching, light sensitivity, pain and soreness, blurred vision, fatigue, and frequent blinking), the DED population was more likely to be bothered and also experienced the symptoms to a greater degree of severity (as reflected by the higher mean scores) than did the normal subjects.

**Table 5:** Conjunctival goblet cell densities at baseline as determined by impression cytology: dry eye versus control populations (all enrolled population – per laboratory protocol)

		All cases (*n* = 217)	All controls (*n* = 67)	*P*-value
Location				
Inferior bulbar	N	168	50	0.4271^a^
	Mean (SD)	86.16 (98.594)	73.85 (86.680)	
	Min–Max	1.20–840.16	3.91–467.64	
Temporal bulbar	N	168	55	0.3643^a^
	Mean (SD)	61.79 (60.778)	75.96 (172.540)	
	Min–Max	1.49–344.40	1.43–1258.31	
	Location comparison^a^	0.0067	0.9380	

^a^ *P*-value taken from a two-sample t-test. Location comparison compares inferior bulbar with temporal bulbar mean values within each treatment group

Additional temporal bulbar samples are represented in control, due to the corresponding inferior bulbar sample with no data

**Table 6:** Survey of symptoms: dry eye disease versus control populations (all enrolled population)

Time Period	Category	Statistic	All cases (*n* = 217)	All controls (*n* = 67)	*P*-value
Past Week					
	Stinging/Burning				
	Experienced, n (%)	None of the time	50 (23.04)	58 (86.57)	<0.0001
		Some of the time	103 (47.47)	8 (11.94)	
		Half of the time	31 (14.29)	0 (0.00)	
		Most of the time	21 (9.68)	1 (1.49)	
		All of the time	8 (3.69)	0 (0.00)	
		Missing	4 (1.84)	0 (0.00)	
	Bothered	N	209	63	<0.0001
		Mean (SD)	1.49 (1.097)	0.19 (0.503)	
	Dryness				
	Experienced, n (%)	None of the time	12 (5.53)	53 (79.10)	<0.0001
		Some of the time	73 (33.64)	10 (14.93)	
		Half of the time	43 (19.82)	3 (4.48)	
		Most of the time	58 (26.73)	0 (0.00)	
		All of the time	28 (12.90)	0 (0.00)	
		Missing	3 (1.38)	1 (1.49)	
	Bothered	N	210	63	<0.0001
		Mean (SD)	2.17 (1.143)	0.24 (0.560)	
	Foreign Body Sensation				
	Experienced, n (%)	None of the time	31 (14.29)	55 (82.09)	<0.0001
		Some of the time	117 (53.92)	11 (16.42)	
		Half of the time	33 (15.21)	0 (0.00)	
		Most of the time	20 (9.22)	1 (1.49)	
		All of the time	13 (5.99)	0 (0.00)	
		Missing	3 (1.38)	0 (0.00)	
	Bothered	N	209	63	<0.0001
		Mean (SD)	1.66 (1.170)	0.25 (0.671)	
	Itching				
	Experienced, n (%)	None of the time	50 (23.04)	47 (70.15)	<0.0001
		Some of the time	88 (40.55)	18 (26.87)	
		Half of the time	36 (16.59)	1 (1.49)	
		Most of the time	32 (14.75)	1 (1.49)	
		All of the time	8 (3.69)	0 (0.00)	
		Missing	3 (1.38)	0 (0.00)	
	Bothered	N	205	63	<0.0001
		Mean (SD)	1.43 (1.168)	0.37 (0.679)	
	Light Sensitivity				
	Experienced, n (%)	None of the time	39 (17.97)	56 (83.58)	<0.0001
		Some of the time	86 (39.63)	11 (16.42)	
		Half of the time	35 (16.13)	0 (0.00)	
		Most of the time	33 (15.21)	0 (0.00)	
		All of the time	21 (9.68)	0 (0.00)	
		Missing	3 (1.38)	0 (0.00)	
	Bothered	N	207	63	<0.0001
		Mean (SD)	1.74 (1.265)	0.21 (0.513)	
	Painful/Sore				
	Experienced, n (%)	None of the time	98 (45.16)	61 (91.04)	<0.0001
		Some of the time	77 (35.48)	6 (8.96)	
		Half of the time	22 (10.14)	0 (0.00)	
		Most of the time	14 (6.45)	0 (0.00)	
		All of the time	2 (0.92)	0 (0.00)	
		Missing	4 (1.84)	0 (0.00)	
	Bothered	N	207	62	<0.0001
		Mean (SD)	1.01 (1.106)	0.11 (0.409)	
	Intermittent Blurred Vision				
	Experienced, n (%)	None of the time	37 (17.05)	57 (85.07)	<0.0001
		Some of the time	104 (47.93)	8 (11.94)	
		Half of the time	44 (20.28)	2 (2.99)	
		Most of the time	18 (8.29)	0 (0.00)	
		All of the time	10 (4.61)	0 (0.00)	
		Missing	4 (1.84)	0 (0.00)	
	Bothered	N	206	61	<0.0001
		Mean (SD)	1.67 (1.175)	0.16 (0.522)	
	Tired/Fatigued Eyes				
	Experienced, n (%)	None of the time	23 (10.60)	43 (64.18)	<0.0001
		Some of the time	108 (49.77)	21 (31.34)	
		Half of the time	32 (14.75)	2 (2.99)	
		Most of the time	39 (17.97)	0 (0.00)	
		All of the time	11 (5.07)	1 (1.49)	
		Missing	4 (1.84)	0 (0.00)	
	Bothered	N	208	62	<0.0001
		Mean (SD)	1.75 (1.109)	0.37 (0.752)	
	Frequent Blinking				
	Experienced, n (%)	None of the time	52 (23.96)	60 (89.55)	<0.0001
		Some of the time	72 (33.18)	6 (8.96)	
		Half of the time	40 (18.43)	1 (1.49)	
		Most of the time	31 (14.29)	0 (0.00)	
		All of the time	18 (8.29)	0 (0.00)	
		Missing	4 (1.84)	0 (0.00)	
	Bothered	N	207	61	<0.0001
		Mean (SD)	1.34 (1.188)	0.10 (0.300)	
	Other				
	Experienced, n (%)	None of the time	55 (25.35)	40 (59.70)	<0.0001
		Some of the time	2 (0.92)	0 (0.00)	
		Half of the time	6 (2.76)	0 (0.00)	
		Most of the time	6 (2.76)	0 (0.00)	
		All of the time	2 (0.92)	0 (0.00)	
		Missing	146 (67.28)	27(40.30)	
	Bothered	N	70	39	0.0015
		Mean (SD)	0.69 (1.314)	0.00 (0.000)	

“Bothered” evaluated by the patient using a 5-point scale where 0 = not at all and 4 = extremely

Symptoms reported as a problem at least some of the time in at least 80% of DED patients included eye dryness (93%), foreign body sensation (84%), light sensitivity (80%), intermittent blurred vision (81%), and tired/fatigued eyes (88%) during the week prior to the baseline study visit (Table 6). In addition, only 15% of DED patients reported that they did not experience intermittent or fluctuating blurring vision at all compared to 73% of normal subjects (Table 7). Mean BCVA (Table 7) of the DED population was 20/24, compared to a mean BCVA of 20/22 in the normal controls (*P* = 0.082). Median BCVA in both populations was 20/20. When asked to what degree they experienced blurring of their vision (0 = none, 1 = mild, 2 = moderate, 3 = severe and 4 = very severe) the two populations diverged significantly, with DED patients reporting a score of 1.6 ± 1.0 (mean ± SD) and median of 2.0, compared to normal subjects reporting a mean score of 0.4 ± 0.7 and a median score of 0.0 (*P* < 0.001). More than half of the DED patients reported that blurring of their vision represented a moderate, severe or very severe problem for them, compared with only 10.5% of normal subjects.

**Table 7:** Measures of visual acuity in dry eye disease and control populations (all enrolled population)

	All cases (*n* = 217)	All controls (*n* = 67)	*P*-value
Best-corrected visual acuity (study eye)			
Mean (SD)	20/24.2 (7.6)	20/22.5 (5.6)	0.0817
Median	20/20	20/20	
Symptom of blurred or fluctuating vision, n (%)			
None	33 (15.2)	49 (73.1)	
Mild	52 (24.0)	10 (15.0)	
Moderate	94 (43.3)	7 (10.5)	
Severe	25 (11.5)	0 (0)	
Very severe	6 (2.8)	0 (0)	
Missing	7 (3.2)	1 (1.5)	
Mean (SD)	1.6 (1.0)	0.4 (0.7)	<0.0001
Median	2.0	0	

Patients in the DED population reported using artificial tears 16.0 ± 12.7 days during the previous month compared to the normal population that averaged 2.27 days of artificial tear use (*P* < 0.0001) (Table 8). Similarly, ointment was used more frequently by the DED patients (2.1 ± 7.1 vs 0) than the normal subjects (*P* = 0.0457). Within the past 3 months, DED patients were more likely to have spent their personal funds on treatments for DED, with an average of $34 (range $0–400), far higher than that reported by the normal population (average of $3 [range $0–30]). Less than half of the DED population described themselves as satisfied or very satisfied with the treatment they were using for their DED.

**Table 8:** Summary of reported treatments for dry eye symptoms: comparison of dry eye disease and control populations

Time Period	Category	Statistic	All cases (*n* = 217)	All controls (*n* = 67)	*P*-value
Past month	Used artificial tears	N	160	49	
		Mean (SD)	16.02 (12.722)	2.27 (6.623)	<0.0001
		Min–Max	0.0–62.0	0.0–30.0	
	Used eye ointments	N	139	45	
		Mean (SD)	2.13 (7.087)	0 (0)	0.0457
		Min–Max	0.0–30.0	0.0–0.0	
Past 3 months	Own money (dollars) spent on treatments for DE (dollars)	N	162	47	
		Mean (SD)	33.56 (57.832)	2.87 (7.854)	0.0004
		Med	15	0	
		Min - Max	0–400	0–30	
	DED symptoms main cause to use medication? n (%)	Eye Drops			
		Antibiotic drops	3 (1.38)	1 (1.49)	0.5044
		Non-prescription	46 (21.20)	6 (8.96)	
		Prescription	5 (2.30)	0 (0.00)	
		Steroids	0(0.00)	0 (0.00)	
		Pills			
		Analgesic/anti-inflammatory (OTC)	4 (1.84)	0 (0.00)	<0.0001
		Analgesics/anti-inflammatory (Rx)	9 (4.15)	0 (0.00)	
		Antidepressants	0 (0.00)	1 (1.49)	
		Antibiotics	0 (0.00)	0 (0.00)	
		Anti-allergy (OTC)	12 (5.53)	3 (4.48)	
		Anti-allergy (Rx)	0 (0.00)	0 (0.00)	
		Other	4 (1.84)	0 (0.00)	
Special procedures, n (%)					
	Punctal occlusion or plugs, n (%)	Have not had	134 (61.75)	42 (62.69)	0.2319
		Less than 1 yr. ago	3 (1.38)	0 (0.00)	
		1–2 years ago	3 (1.38)	0 (0.00)	
		3 or more yrs. ago	8 (3.69)	0 (0.00)	
	Eye lid surgery, n (%)	Have not had	138 (63.59)	41 (61.19)	0.6136
		Less than 1 yr. ago	0 (0.00)	0 (0.00)	
		1–2 yrs. ago	1 (0.46)	0 (0.00)	
		3 or more yrs. ago	8 (3.69)	1 (1.49)	
	Overall satisfaction with treatment, n (%)	Currently not using treatments	37 (17.05)	37 (55.22)	<0.0001
		Overall dissatisfied	0 (0.00)	0 (0.00)	
		Very dissatisfied	11 (5.07)	0 (0.00)	
		Somewhat dissatisfied	28 (12.90)	1 (1.49)	
		Neutral	32 (14.75)	5 (7.46)	
		Somewhat satisfied	36 (16.59)	1 (1.49)	
		Very satisfied	19 (8.76)	4 (5.97)	
		Missing result	54 (24.88)	19 (28.36)	

*OTC* Over-the-counter, *Rx* prescription

For artificial tear and eye ointment use, the number of days used in the past 1 month is presented

*P*-values for continuous endpoints taken from two sample *t*-test. *P*-values from categorical endpoints taken from chi-squared test

DED patients reported more frequent visits with both optometrists and ophthalmologists but not with other health care professionals during the prior year (*P* ≤ 0 .0001) (Table 9). A higher percentage of the normal subjects reported themselves to be currently employed than was the case among the DED population (60% vs 47%,  *P*= 0.0699). The two populations were significantly different in how they reported being impacted by health problems. Using a scale of 0 to 10, in which 0 equals “no effect” and 10 equals “completely prevented me from performing the activity”, in both productivity ( *P* = 0.0003) and in non-work-related “regular activities” ( *P *< 0.0001), DED patients were more likely than normal subjects to report being negatively impacted by their health problems (Table 10).

**Table 9:** Reported doctor visits in prior year: comparison between dry eye disease and control populations

Time Period	Category	Statistic	All cases (*n* = 217)	All controls (*n* = 67)	*P*-value
Past Year	Doctor visits related to dry eye				
		Mean (SD)	0.10 (0.439)	0.00 (0.000)	
		Min–Max	0.0–4.0	0.0–0.0	
		1 Visit, n (%)	7 (3.23%)	0 (0.00)	
		>1 Visit, n (%)	3 (1.38%)	0 (0.00)	
	Ophthalmologist	N	159	47	<0.0001
		Mean (SD)	0.64 (1.002)	0.00 (0.000)	
		Min–Max	0.0–6.0	0.0–0.0	
		1 Visit, n (%)	35 (16.13%)	0 (0.00)	
		>1 Visit, n (%)	27 (12.44%)	0 (0.00)	
	Optometrist	N	158	47	0.0013
		Mean (SD)	0.37 (0.919)	0.00 (0.000)	
		Min–Max	0.0–6.0	0.0–0.0	
		1 Visit, n (%)	17 (7.83%)	0 (0.00)	
		>1 Visit, n (%)	14 (6.45%)	0 (0.00)	
	Rheumatologist	N	154	46	0.4085
		Mean (SD)	0.05 (0.425)	0.00 (0.000)	
		Min–Max	0.0–5.0	0.0–0.0	
		1 Visit, n (%)	3 (1.38%)	0 (0.00)	
		>1 Visit, n (%)	1 (0.46%)	0 (0.00)	
	Mental health counselor	N	154	46	0.5860
		Min–Max	0.0–5.0	0.0–0.0	
		1 Visit, n (%)	0 (0.00)	0 (0.00)	
		>1 Visit, n (%)	1 (0.46%)	0 (0.00)	
	Other	N	86	32	0.2323
		Mean (SD)	0.08 (0.382)	0.00 (0.000)	
		Min–Max	0.0–2.0	0.0–0.0	
		1 Visit, n (%)	1 (0.46%)	0 (0.00)	
		>1 Visit, n (%)	3 (1.38%)	0 (0.00)

**Table 10:** Comparison of work productivity and activity impairment questionnaire at baseline between dry eye disease and control populations (all enrolled population)

Question	All cases (*n* = 217)	All controls (*n* = 67)	*P*-value
Currently employed, n (%)			
Yes	100 (46.76%)	40 (59.70%)	0.0699
No	114 (53.27%)	27 (40.30%)	
Hours missed work due to health			
N	106	42	
Mean (SD)	0.48 (2.846)	0.19 (1.234)	0.5248
Min–Max	0–25	0–8	
Hours missed work due to other reasons			
N	106	42	
Mean (SD)	2.02 (5.174)	2.30 (6.098)	0.7776
Min–Max	0–40	0–30	
Hours actually worked			
N	109	42	
Mean (SD)	30.87 (16.202)	31.04 (17.603)	0.9554
Min–Max	0–62	0–60	
Productivity affected by health problems			
N	108	42	
Mean (SD)	1.63 (2.538)	0.17 (0.660)	0.0003
Min–Max	0–10	0–3	
Regular activities affected by health problems			
N	200	66	
Mean (SD)	1.83 (2.280)	0.59 (1.617)	<0.0001
Min–Max	0–10	0–9	

Health problem productivity was evaluated by the patient using an 11-point scale where 0 = no effect and 10 = completely prevented me from working. Health problem activities were evaluated by the patient using an 11 point scale where 0 = no effect and 10 = completely prevented me from doing my daily activities

*P*-values for continuous endpoints taken from two sample *t*-tests

*P*-values from categorical endpoints taken from chi-squared test

The levels of inflammatory cytokines measured in the DED patients and normal subjects are listed in Table 11. While there was no statistically significant difference in the mean values of any of the tested cytokines between the two groups, the upper range of many of these cytokines were considerably higher in the DED group. An analysis to determine correlations in levels of these various markers demonstrated that, in the DED population, the strongest correlations were between IL-8 and IL-6 (correlation coefficient 0.806; *P* < 0.0001), IL-6 and EGF (correlation coefficient 0.767; *P* < 0.0001) and between IL-8 and EGF (correlation coefficient 0.756; *P* < 0.0001) (Table 12). In the normal control subjects (Table 13), IL-8 levels similarly correlated, but less strongly, with EGF (correlation coefficient 0.576; *P* < 0.0001) and with IL-6 (correlation coefficient 0.707; *P* < 0.0001). The data were analyzed to determine if the measured levels of inflammatory cytokines correlated with specific clinical signs. In evaluating DED patients and controls, corneal staining correlated with IFNγ (*P* = 0.0358, r^2^ = 0.144) and TNF-α (*P* = 0.0405, r^2^ = 0.141).

**Table 11:** Comparison of tear cytokine levels at baseline between dry eye disease and control populations (all enrolled population)

Variable pg/mL/mm		All cases (*n* = 217)	All controls (*n* = 67)	*P*-value
EGF	N	164	51	
	Mean (SD)	2074.88 (3739.842)	1724.86 (2632.770)	0.5348
	Min-Max	0.0–21,938	0.0–12,827	
IFN-γ	N	164	51	
	Mean (SD)	23.16 (125.014)	18.77 (57.550)	0.8083
	Min-Max	0.0–1168.4	0.0–322.5	
IL-1α	N	164	51	
	Mean (SD)	214.58 (404.996)	159.45 (223.319)	0.3543
	Min-Max	0.0–3244.0	0.0–1241.3	
IL-1β	N	164	51	
	Mean (SD)	55.89 (155.754)	25.02 (36.211)	0.1626
	Min-Max	0.0–1580.9	0.0–194.6	
IL-2	N	164	51	
	Mean (SD)	7.79 (25.646)	4.07 (7.751)	0.3091
	Min-Max	0.0–253.4	0.0–33.3	
IL-4	N	164	51	
	Mean (SD)	124.53 (433.605)	152.13 (331.354)	0.6765
	Min-Max	0.0–3229.7	0.0–1913.5	
IL-6	N	164	51	
	Mean (SD)	147.45 (476.145)	104.72 (290.994)	0.5451
	Min-Max	0.0–3996.7	0.0–1801.2	
IL-8	N	164	51	
	Mean (SD)	722.86 (1617.582)	563.38 (769.371)	0.4974
	Min-Max	0.0–15,528	0.0–3240.8	
IL-10	N	164	51	
	Mean (SD)	29.06 (112.567)	34.11 (104.294)	0.7765
	Min-Max	0.0–1175.0	0.0–727.5	
IL-13	N	164	51	
	Mean (SD)	48.48 (181.660)	19.36 (44.051)	0.2586
	Min-Max	0.0–1662.7	0.0–277.6	
IL-17	N	164	51	
	Mean (SD)	15.77 (91.369)	16.17 (67.701)	0.9768
	Min-Max	0.0–911.4	0.0–394.7	
IP-10	N	164	51	
	Mean (SD)	42,229.7 (61,361.73)	33,137.9 (37,534.33)	0.3182
	Min-Max	0.0–449,861	0.0–144,295	
TNF-α	N	164	51	
	Mean (SD)	13.30 (66.885)	5.95 (17.235)	0.4387
	Min-Max	0.0–642.1	0.0–86.1	

*EGF* Epidermal growth factor, *IFN-γ* Interferon gamma, *IL* Interleukin, *IP-10* Interferon-gamma-inducible protein 10, *TNF-α* Tumor necrosis factor alpha

*P*-values taken from two sample *t-*test

**Table 12:** Correlations among cytokine levels at baseline in dry eye disease patients (all enrolled population)

	EGF	IFN-γ	IL-1α	IL-1β	IL-2	IL-6	IL-8	IL-17	IP-10	TNF-α
IFN-γ	0.30400 < 0.0001	–								
IL-1α	0.23297 0.0027	0.32068 < 0.0001	–							
IL-1β	0.51640 < 0.0001	0.41754 < 0.0001	0.66743 < 0.0001	–						
IL-2	0.25249 0.0011	0.49808 < 0.0001	0.16664 0.0329	0.42179 < 0.0001	–					
IL-6	0.76740 < 0.0001	0.35501 < 0.0001	0.33220 < 0.0001	0.57020 < 0.0001	0.23707 0.0022	–				
IL-8	0.75670 < 0.0001	0.28040 0.0003	0.24529 0.0015	0.40306 < 0.0001	0.27415 0.0004	0.80562 < 0.0001	–			
IL-17	0.31044 < 0.0001	0.45138 < 0.0001	0.27422 0.0004	0.30745 < 0.0001	0.17318 0.0266	0.38157 < 0.0001	0.26181 0.0007	–		
IP-10	0.58512 < 0.0001	0.15471 0.0479	0.33144 < 0.0001	0.38843 < 0.0001	0.06706 0.3936	0.64034 < 0.0001	0.69808 < 0.0001	0.21564 0.0056	–	
TNF-α	0.37737 < 0.0001	0.58537 < 0.0001	0.27896 0.0003	0.44958 < 0.0001	0.56420 < 0.0001	0.49181 < 0.0001	0.40005 < 0.0001	0.36543 < 0.0001	0.26294 0.0007	–

*EGF* Epidermal growth factor, *IFN-γ* Interferon gamma, *IL* Interleukin, *IP-10* Interferon-gamma-inducible protein 10, *TNF-α* Tumor necrosis factor alpha

Correlation coefficients and *P*-values (H_0_:Rho = 0) are presented within each cell

**Table 13:** Correlations among cytokine levels in control subjects at baseline (all enrolled population)

	EGF	IFN-γ	IL-1α	IL-1β	IL-2	IL-6	IL-8	IL-17	IP-10	TNF-α
IFN-γ	0.28163 0.0431	–								
IL-1α	0.04026 0.7769	0.16494 0.2426	–							
IL-1β	0.29569 0.0333	0.05001 0.7248	0.51570 < 0.0001	–						
IL-2	0.20250 0.1500	0.19394 0.1683	0.27698 0.0468	0.47065 0.0004	–					
IL-6	0.40299 0.0031	0.08991 0.5262	0.42265 0.0018	0.51464 < 0.0001	0.38000 0.0055	–				
IL-8	0.57592 < 0.0001	0.09937 0.4834	0.25712 0.0657	0.32132 0.0202	0.25218 0.0713	0.70706 < 0.0001	–			
IL-17	0.21875 0.1192	0.29428 0.0342	0.31177 0.0245	0.32902 0.0172	0.09588 0.4989	0.35271 0.0103	0.26003 0.0626	–		
IP-10	0.59353 < 0.0001	0.09289 0.5125	0.28451 0.0409	0.25914 0.0636	0.18776 0.1826	0.42547 0.0017	0.55959 < 0.0001	0.20005 0.1551	–	
TNF-α	0.18272 0.1948	0.29141 0.0361	0.13719 0.3321	0.32506 0.0187	0.55169 < 0.0001	0.57917 < 0.0001	0.34301 0.0128	0.36613 0.0076	0.15190 0.2824	–

*EGF* Epidermal growth factor, *IFN-γ* Interferon gamma, *IL* Interleukin, *IP-10* Interferon-gamma-inducible protein 10, *TNF-α* Tumor necrosis factor alpha

Correlation coefficients and P-values (H_0_:Rho = 0) are presented within each cell

## Discussion

Our understanding of DED has evolved substantially in recent decades. In 1995, a National Eye Institute-sponsored workshop concluded that DED is a disorder of the tear film due to tear deficiency or excessive evaporation, which causes damage to the interpalpebral ocular surface and is associated with symptoms of ocular discomfort [15]. In 2007, a DED Workshop defined DED as a multifactorial disease of the tears and ocular surface resulting in symptoms of discomfort, visual disturbance and tear film instability with potential damage to the ocular surface. It is accompanied by increased osmolarity of the tear film and inflammation of the ocular surface [17]. This change from “disorder” to “disease” and the appreciation of underlying inflammation highlight the enhanced understanding of the pathology in these patients and underscores the importance of our understanding its natural history.

Our baseline findings demonstrate major differences between the patients and the normal control subjects. All subjects included in the study with DE were aqueous deficient with or without an evaporative component. Although our study population consisted only of patients with mild-to-moderate DE, the study population reported major problems with blurred vision. The median acuities in both patients and normals were 20/20, yet the majority of patients reported moderate-to-severe symptoms of blurred vision. Presumably reflecting the importance of the precorneal tear film, this finding suggests that complaints about blurring may be a helpful metric for distinguishing DED patients from normals. This finding also reflects the importance of a thorough assessment of symptoms beyond visual acuity in evaluating DED patients and understanding the potential impact of DE on vision. The proposed mechanism for blurred vision involves ocular surface damage in the overlying optical zone (central corneal regions) which is associated with increased higher-order aberrations and increased corneal backward light scattering [18]. The impact of DED on vision also has functional implications to daily activities and quality of life. Studies have shown that reading rates are significantly reduced in DE patients vs control subjects, and deteriorating visual function correlated with DED severity [19, 20]. This suggests that reduced visual function associated with DED can interfere with daily visual activities and quality of life.

The baseline goblet cell density in the temporal quadrant of DED patients tended to be lower than in the control group, but the difference did not achieve statistical significance. There was no difference in the goblet cell density in the inferior quadrant between diseased and normals, consistent with the hypothesis that the exposed temporal conjunctiva may be more susceptible to the effects of DE. Lack of statistical significance in the difference in the temporal conjunctiva can be explained by the relatively mild-to-moderate degree of disease in this patient population. The goblet cell densities measured in both the DED and normal populations at baseline are lower than those reported in a younger population of normal patients [21], suggesting that older individuals may have lower goblet cell densities than do younger individuals.

The concept that DED reflects an underlying inflammation of the ocular surface (localized autoimmune disorder) is relatively recent [22]. Consistent with the concept of underlying inflammation is the finding that several inflammatory cytokines were found to be elevated in the DED group compared to the normal subjects, even though the mean values between the two groups were not statistically different. This supports the notion of dry eye disease as a continuum characterized by a complex interplay of pathogenic mechanisms with inflammation increasingly involved as dry eye disease progresses. Whether inflammation is the inciting factor of dry eye or is a consequence of other factors, the baseline findings reported here suggest that the majority of moderate dry eye subjects have underlying inflammation on the ocular surface.

Elevated levels of certain cytokines were correlated with physical findings typical of DE. These findings are consistent with the literature, which suggests tear hyperosmolarity is specifically associated with higher levels of the proinflammatory cytokine IFN-γ, which correlates with key clinical parameters of DED [23]. There is also evidence that IL-17, TNF-α, and IL-6 are significantly elevated in DED compared with controls, and that a significant correlation exists between IL-6 and tear film break -up time in DE subjects [24]. In this study, the large variability in tear cytokine concentrations in both groups might be attributed to reflex tearing in some of the samples. Future studies could account for reflex tearing and assess cytokines relative to total tear protein.

A limitation of this study was that eligibility criteria for the control subjects may not have been sufficient to exclude mild evaporative dry eye disease. Additional tests related to tear film stability or meibomian gland activity might have been beneficial in this cohort. However, the inclusion/exclusion criteria for control subjects still excluded any subjects who had any evidence of corneal staining, conjunctival staining, or regular symptoms of DED*.* The pipetting error range of ±1 μL could also be interpreted as a potentially significant range (~20%) given the small volume (10 μL) that was prepared for the cytokine analysis. The study would also have benefited by analyzing baseline findings by region to determine potential differences due to climatic variability.

## Conclusions

The baseline data generated in this study demonstrate that relatively mild stages of DED may have substantive negative impacts on patients’ lives, including blurred vision, more frequent visits to eye care professionals, productivity, as well as regular activities. The results of the longitudinal study may provide a service to both DED patients and treating physicians as we may be able to better characterize the findings that are more predictive of disease progression which may warrant earlier therapeutic interventions with hopefully superior outcomes.

## Abbreviations

BCVA: best-corrected visual acuity
DED: dry eye disease
EGF: epidermal growth factor
IFN: interferon
IL: interleukin
IP: inducible protein
ITF: International Task Force
Max: maximum
Min: minimum
OSDI: Ocular Surface Disease Index
PROOF: The Progression of Ocular Findings
SD: Standard deviation
TNF: Tumor necrosis factor
